# Epidemiological study on behavioural and emotional problems in developmental age: prevalence in a sample of Italian children, based on parent and teacher reports

**DOI:** 10.1186/1824-7288-40-19

**Published:** 2014-02-18

**Authors:** Antonella Gritti, Carmela Bravaccio, Simona Signoriello, Filomena Salerno, Simone Pisano, Gennaro Catone, Ciro Gallo, Antonio Pascotto

**Affiliations:** 1 Faculty of Education Science, University Suor Orsola Benincasa of Naples, Napoli, Italy; 2 Department of Translational Medical Sciences, University Federico II of Naples, Via Pansini 5, 80131 Naples, Italy; 3 Department of Medicine and Public Health, Second University of Naples, Naples, Italy; 4 Department of Mental and Physical Health and Preventive Medicine, University of Naples SUN, Naples, Italy

**Keywords:** Behavioral problems, CBCL, Emotional well-being

## Abstract

**Background:**

The aim of this study is to examine the prevalence of behavioural and emotional problems in a sample of school children living in Campania, a region of South Italy.

**Methods:**

The Child Behavior Checklist (CBCL) Parent Report Form (PRF) and the CBCL Teacher Rating Form (TRF) were administered to parents and teachers of a sample of school children aged 8-9 yr.

**Results:**

The subjects (SS) eligible for the study were 3072. In 2137 (69.5%) cases parents returned the envelopes back. 1228 (57.4%) subjects were excluded because of lack of signed consensus, unfilled or incomplete forms. Parents reported children’s behavioural or emotional *Total Problems* in 14.7% of the SS. (5.2% borderline, 9.5% clinical), *Internalizing Problems* in 18.5% (8.0% borderline, 10.5% clinical), and *Externalizing Problems* in 8.5% (3.8% borderline, 4.7% clinical) respectively. At the *Competence Scale* of CBCL more than 2/3 of the sample show high rate for *Total Competence Problem* (24.3% *borderline,* 47.3% *clinical*.) Teachers reported 8.7% of SS having *Total Problems,* (4.3% borderline, 4.4% clinical), *Internalizing* problems were detected in 13.3% of the sample (4.9% borderline and 8.4% clinical), while *Externalizing* problems were reported for 9.6% of SS (4.1% borderline and 5.5% clinical). In the sub-scale of *Academic Performances* teachers report a high number of subjects with problems, 18.7%, whose 4.3% had a “borderline” score, and 14.4% had a “clinical” score.

**Conclusion:**

Concerning *Total Problems* (clinical and borderline SS, 14.7% as reported by parents, 8.7% as reported by teachers) we obtained a prevalence similar to that reported in the rest of the country, with differences in gender (males 13.2%, females 16.0% as reported by parents; males 7.4%, females 9.7% as reported by teachers). The difficulties in social and relationship competencies area were higher (4/10 children). This datum should be cautiously evaluated because the possible inadequacy of CBCL competences scale.

## Background

Epidemiological studies of psychiatric disorders in children and adolescents have been conducted in several European Countries [1-8].

Results showed large differences in prevalence across studies, with estimated rates, for emotional and behavioural problems in children and adolescents, between 9% and 20%.

Epidemiological studies using screening instruments allow to assess the probability of mental health problems; DSM or ICD diagnosis need to be assessed throughout clinician rated specific interviews. Child Behavior Checklist (CBCL) is a widely used screening instrument and it was found to be powerful and very sensitive in youths [4].

In Italy, Frigerio et al., using CBCL, estimated a prevalence of mental disorders in a random sample of 3418 students aged 10-14 years old, living in urban areas of Center and Northern Italy. Results showed a prevalence of CBCL caseness and DSM-IV diagnosis of 9.8% and 8.2% respectively (with DSM-IV emotional disorders more frequent than externalizing ones) [8], whereas no data are available on the prevalence of mental disorders in children or adolescents in Southern Italy. To overcome this, in 2005 we promoted a two stage project focused on childhood Mental Health in the Campania Region, in Southern Italy and in this paper we present the results of the first stage of the study. We aimed to estimate the risk for emotional and behavioral problems in 8 to 9 years-old students attending primary school in Campania, as reported by parents and teachers^a^. Based on previous Danish paper [4], we investigated this age-frame because we were interested in pre-adolescent population, furthermore children that have attended the primary school since two years were well known by teachers and this allowed us to narrow potential confusing variables. A previous Danish paper reported teachers’ assessment to be valid, especially on externalizing disorder [9].

We tested the specific hypothesis that rate of Total Problems, Internalizing Problems and Externalizing Problems might be higher in South Italy sample of children than the one reported by Frigerio [8].

## Methods

### Subjects and procedures

Study subjects (SS) were 8 to 9 year-old children attending 19 primary schools in four Campania Region provinces (Southern Italy), who agreed to participate.

The study was supported by the Campania Region and was conducted in collaboration between the Second University of Naples, the Regional School Bureau and the community medical structures (ASL). Research protocol and procedures to obtain parental consent were reviewed by Regional School Bureau. The research was approved by the Institutional Review Board of Medicine and Surgery, Polyspecialistic Department of the Second University of Naples and was performed according to the Helsinki Declaration.

Several public meetings were held by the research team at every school-district with the participation of the school principals, teachers, community child psychiatrists, and parents of the eligible students. The aim of these meetings was to present the goals of the projects and to ask for willingness to participate. Parents were involved in a further meeting that was held at each school. They were fully informed, verbally and trough a written form, regard aims and phases of the study, data confidentiality and instruments applied. Parents were asked to provide a written informed consent for their child to be enrolled in the study and for child’s teacher to fulfill the *Child Behaviour Checklist 6-18-Teacher report Form (CBCL -TRF).*

*Child Behaviour Checklist 4-18 -Parent Report Forms (CBCL-PRF*) was hand over to each parent in a sealed envelope, together with a detailed information sheet and a consent form. Parents had to return the questionnaire and the consent forms in a sealed envelope, within two weeks. The same method was followed for teachers. Parents and teacher were asked to answer the questionnaires in the same time frame.

Children with certified intellectual or developmental disabilities (as know from territorial medical structures specifically contacted for this reason) were excluded from the study because CBCL is not a reliable instrument in these populations.

Each family was informed about the results of their own child CBCL. The parents whose children were within the “clinical” or the “borderline” range, were invited to participate to the phase 2 of the project.

3072 subjects were contacted. In 2137 (69.5%) cases parents returned the envelopes back. 1228 subjects were excluded because of lack of signed consent, unfilled or incomplete forms. 909 children (469 males, 52% and 440 females, 48%) were assessed by at least one parent. Mean age (± standard deviation) was equal to 8.6 ± 0.5 years; with similar values in males and females (8.6 ± 0.5 and 8.6 ± 0.6 years, respectively). Both parents provided information for 510 (56%) children. 1390 children (626 males, 45% and 764 females, 55%) were assessed by teachers. 471 subjects were fully screened by both the teacher and at least one parent.

Data on socio-economic status were not recorded due to poor collaboration of schools. It was specifically required by parents’ council to avoid providing these informations. 96% of children were white Caucasian, whereas 4% were Asian/African.

### Measures

The Achenbach scales CBCL-PRF and CBCL-TRF [10,11] were used to obtain standardized assessments of children’s behaviour for screening purposes.

The CBCL-PRF scale is a standardized questionnaire, widely used in epidemiological and clinical studies, that assess behaviour problems and competences of children aged 4-18 years. It consists of 113 items and is filled in by parents using a 0-1-2 Likert scale (0, not true; 1, somewhat or sometimes true; and 2, very or often true).

Eight sub-scales (or Syndrome Scales) are derived: Withdrawn/Depressed, Somatic Complaints, Anxious/Depression, Social Problems, Thought Problems, Attention Problems, Rule-breaking Behaviour, Aggressive Behaviour, as well as a scale for Sex Problems.

Three more inclusive scales ensue from combining some sub-scales: ‘Internalizing’, ‘Externalizing’ and ‘Total Problems’ score. Three scales assess the sphere of competence: Activities, Social Function and School. A Total Competence score is obtained by summing up these scales.

Scores are transformed into a T-score, based on age and gender norms, and three categories arise: Normal, Borderline and Clinical children.

The CBCL-TRF has been designed to be completed by teachers of students aged between 6 and 18 years, to assess their behaviors, academic performances and adaptive competences. In present paper, it was required that each child had to be known by the teachers since at least two years.

We used the Italian version of the CBCL-PRF and CBLL-TRF [12,13]. No normative Italian data on CBCL were available at the time of this research. Nevertheless, the psychometric properties of CBCL Italian version and the validity and reliability of the CBCL for use in Italian population had been shown [3,8]; internal consistency was reported to be good, ranging between > .78 for Total Problems and the two broadband scales, and > .65 for most narrowband scales. Inter-rater agreement was reported to be low to moderate depending on age and sex, with higher concordance rating boys. CBCL score were determined using Achenbach’s normative data [10,11].

### Statistical analysis

Every single form was analyzed with the ADM software (Assessment Data Manager 6.10, by T.M. Achenbach and L.A. Rescorla).

CBCL-PRF categories were derived as follows: children were labeled as Normal (N) if both parents (or the sole responding parent) reported N, Clinical (C) if at least one parent reported C, and Borderline (B) otherwise. Differences of scores by gender were assessed by exact chi-square test. When the responses of both parents were available, symmetry of the responses within the matched pairs was tested by Mc Nemar’s test, combining together B and C categories. The degree of agreement was estimated by Kappa statistics, which ranges from zero (lack of any improvement beyond the chance) to 1 (perfect agreement between assessors). As a rule of thumb a good agreement is observed when Kappa >0.6.

All analysis were performed with R statistical software, version 2.12.2 (The R Foundation for Statistical Computing) [14] and StatXact 7 software (Cytel Inc. 2005).

## Results

### CBCL parent report

Out of the 909 children who were assessed by at least one parent, 45% and 55% were 8- and 9-year old, respectively. Both parents provided assessment for 510 (56.1%) children; in 399 cases, (43.8%) when only one parent responded, mother was largely prevailing (86.%).

Distribution of subjects within *normal*, *borderline* or *clinical* ranges as reported by parents is reported in Table 1.

**Table 1:** CBCL PRF (909SS)

	**Total**			**Males**			**Females**		
	**N**_**%**_	**B**_**%**_	**C**_**%**_	**N**_**%**_	**B**_**%**_	**C**_**%**_	**N**_**%**_	**B**_**%**_	**C**_**%**_
**COMPETENCES**									
Total competences	28.4	24.3	47.3	24.9	26.4	48.6	31.6	22.3	46.0
*Activity*	44.9	34.2	20.9	49.9	25.5	24.6	40.4	42.1	17.5
*Social functions*	90.6	6.4	3.0	93.3	5.3	1.4	88.0	7.5	4.5
*School*	97.7	1.8	0.5	97.2	1.9	0.9	98.3	1.7	0.0
**PROBLEMS**									
Syndromic scales									
*Withdrawal*	96.5	2.3	1.2	97.7	1.4	0.9	95.4	3.1	1.5
*Somatic complaints*	95.3	3.2	1.6	95.8	1.9	2.3	94.8	4.4	0.9
*Anxiety/Depression*	94.0	3.3	2.7	95.6	2.8	1.6	92.6	3.7	3.7
*Social problems*	92.1	5.5	2.4	93.3	3.5	3.3	91.0	7.4	1.5
*Thought problems*	97.5	2.1	0.3	97.9	1.9	0.2	97.2	2.4	0.4
*Attention problems*	94.0	3.5	2.5	94.9	3.0	2.1	93.2	3.9	2.8
*Delinquent behavior*	96.5	2.8	0.7	96.3	3.5	0.2	96.7	2.2	1.1
*Aggressive behavior*	96.2	2.6	1.2	96.7	2.3	0.9	95.6	2.8	1.5
Internalizing	81.5	8.0	10.5	86.0	6.0	7.9	77.3	9.8	12.9
Externalizing	91.4	3.8	4.7	93.7	2.6	3.7	89.3	5.0	5.7
Total problems	85.4	5.2	9.5	86.7	6.0	7.2	84.1	4.4	11.6

*N*, Normal; *B*, Borderline; *C*, Clinical.

Parents reported children’s behavioural or emotional problems in 14.7% (5.2% borderline, 9.5% clinical) on *Total Problems* (males 13.2%, females 16.0%), in 18.5% (8.0% borderline, 10.5% clinical) on *Internalizing*, (males 13.9%, females 22.7%) and in 8.5% (3.8% borderline, 4.7% clinical) on *Externalizing* scales (males 6.3%, females 10.7%). Percentages of problems in single subscales never exceeded 8%.

Conversely, an astonishingly 71.6% was reported on the *Competence Scale* (24.3% *borderline,* 47.3% *clinical*), mostly due to the *Activity* subscale.

Relevant differences between gender were reported for *Activity* and *Social functions* subscales and Internalizing problems.

### Agreement between mother and father

Agreement between mother and father in the 510 children who were assessed by both parents is shown in Table 2. Overall the agreement of responses between parents, as measured by Kappa value, was fair, being greater than 0.6 just for Activity and Attention scores. However no systematic differences were observed between parents, but for Somatic Complaints, that were more easily reported by Mother (p < 0.001).

**Table 2:** Agreement between mother and father (510 SS)

	**n.**	**n.**	**n.**	**n.**			
	**M**_**N **_**F**_**N**_	**M**_**BC **_**F**_**BC**_	**M**_**N **_**F**_**BC**_	**M**_**BC **_**F**_**N**_	**Tot**	**P value cond**	**P value**
***COMPETENCES***							
Total competence	128	223	40	36	427	0.731	< 0.001
*Activity*	199	181	44	38	462	0.5811	< 0.001
*Social functions*	421	13	18	13	465	0.4731	< 0.001
*School*	478	3	4	6	491	0.7539	< 0.001
***PROBLEMS***							
Syndromic scales							
*Withdrawal*	481	7	5	7	500	0.7744	< 0.001
*Somatic complaints*	475	8	2	15	500	0.00235	< 0.001
*Anxiety/Depression*	465	13	10	12	500	0.8318	< 0.001
*Social problems*	461	14	12	13	500	1	< 0.001
*Thought problems*	487	4	4	5	500	1	< 0.001
*Attention problems*	467	20	7	6	500	1	< 0.001
*Deliquent behavior*	483	4	5	8	500	0.5811	< 0.001
*Aggression bheavior*	478	8	8	6	500	0.7905	< 0.001
Internalizing	407	45	18	30	500	0.1114	< 0.001
Externalizing	459	19	9	13	500	0.5235	< 0.001
Total problem	423	42	15	20	500	0.4996	< 0.001

*M*, Mother; *F*, Father; *N*, Normal; *B*, Bordeline; *C*, Clinical.

### CBCL Teacher’s report

Tables 3 and 4 provides information on distribution of subjects within *normal*, *borderline* or *clinical* ranges as reported by teachers. *Internalizing problems* were reported in 13.3% of children (8.4% clinical and 4.9% borderline) with a highly significant difference by gender (males 11.1%, females 15.1%). Conversely, *Total Problems* and *Externalizing problems* were reported in 8.7% and 9.6%, respectively, without difference between genders. Highly significant differences between gender were also reported for anxiety problems, that overall were reported in 12% (3% clinical and 9% borderline) of children. It is worth noting the high prevalence (14%) of clinical difficulties reported by teachers for the *Academic performance* subscale.

**Table 3:** CBCL TRF (1390 SS.)

	**Total**			**Males**			**Females**		
	**N%**	**B%**	**C%**	**N%**	**B%**	**C%**	**N%**	**B%**	**C%**
Total items	81.5	9.9	8.6	75.3	14.3	10.4	86.6	6.4	7.1
*Anxiety/Depressed*	94.6	3	2.4	96.8	1.9	1.3	92.8	3.8	3.3
*Withdrawn/Depressed*	93.5	3.7	2.8	94.2	3.2	2.6	92.8	4.1	3.1
*Somatic complaints*	96	3.1	0.9	97.1	2.6	0.3	95.1	3.6	1.3
*Social problems*	95	3.1	1.9	94.8	3.7	1.4	95.1	2.7	2.3
*Thought problems*	98	0.9	1.1	98.7	1	0.3	97.3	0.9	1.7
*Attention problems*	97.7	1.6	0.7	98.2	1	0.8	97.2	2.1	0.7
*Rule-breaking behavior*	95.1	3.5	1.5	95.8	3.2	1	94.4	3.7	1.9
*Aggressive behavior*	96.1	2.5	1.4	97.4	1.6	1	95	3.3	1.7
*Academic performance*	81.3	4.3	14.4	80.3	5.7	13.9	82.1	3.1	14.9
Internalizing problems	86.7	4.9	8.4	88.9	5.6	5.5	84.9	4.2	10.9
Externalizing problems	90.4	4.1	5.5	91.1	3.9	5	89.8	4.2	6
Total problems	91.3	4.3	4.4	92.6	4	3.4	90.3	4.5	5.2
*Affective problems*	95.3	3.1	1.6	95.5	3.4	1.1	95.1	2.9	2
*Anxiety problems*	88.5	8.6	2.9	90.8	8.1	1.1	86.6	9	4.4
*Somatic problems*	97.4	1.9	0.7	98.1	1.8	0.2	96.8	2	1.2
*ADHD*	97.2	2	0.8	98.2	1	0.8	96.4	2.8	0.8
*Oppositional defiant problems*	97.1	1.8	1.1	97.6	1.4	1	96.7	2.1	1.2
*Conduct problems*	96.7	1.6	1.7	97.9	0.8	1.3	95.8	2.3	2

*N*, Normal; *B*, Borderline; *C*, Clinical.

**Table 4:** Agreement between parents and teaches reports (471 SS)

	**n**	**n**	**n**	**n**	**n**		
	**P**_**N **_**T**_**N**_	**P**_**BC **_**T**_**BC**_	**P**_**N **_**T**_**BC**_	**P**_**BC **_**T**_**N**_	**Tot**	**P value cond**	**P value**
**COMPETENCES**							
Total competences	91	51	14	230	386	< 0.001	0.2881
*School*	348	1	61	4	414	< 0.001	1
**PROBLEMS**							
Syndromic scales							
*Withdrawn*	380	3	28	12	423	0.01659	0.08881
*Rule-breaking behavior*	394	3	19	7	423	0.02896	0.01159
*Aggerssive behavior*	394	2	15	12	423	0.7011	0.1041
*Social problems*	383	7	10	23	423	0.03508	< 0.001
*Thought problems*	410	1	3	9	423	0.146	0.09158
*Attention problems*	391	2	5	25	423	0.0003249	0.06758
*Anxious/Depressed*	381	3	19	20	423	1	0.1097
*Somatic complaints*	394	0	8	21	423	0.02412	1
Total problems	344	11	22	45	422	0.006741	0.001636
Internalizing	299	16	44	63	422	0.08135	0.1068
Esternalizing	364	9	28	21	422	0.3916	< 0.001

*P*, Parent; *T*, Teacher; *N*, Normal; *B*, Borderline; *C*, Clinical.

## Discussion

In this paper, we reported the results of a population–based screening study to estimate the risk for emotional and behavioral problems in 8 to 9 year-old students attending primary school in Campania Region, as reported by parents and teachers. To our knowledge this is the first epidemiological survey in Southern Italy. The PrISMA study [8], indeed, focused on children aged 10-14 years old living in urban areas of Center and Northern Italy.

In our study, according to parents, about a tenth of children have showed clinical emotional or behavioural problems and a further 5% presented subclinical (borderline) problems. Compared with other findings [8], *Total Problems* have a similar prevalence ratio. A high percentage of students with *Internalizing* (11%) or *Externalizing* (5%) problems were found. As in Frigerio’s study, it was confirmed that *Internalizing* problems are more frequent than *Externalized* ones; anyway, as hypothesized, percentage of externalizing problems was higher, consistent with studies from other countries [14]. Regarding gender differences, our results emphasize that both parents and teachers are more likely to report that females had more internalizing problems and more difficulties in the activity subscale, while only for the parents, differences in gender were reported also in social subscale (prevalence in females); this reflects a trend observed for adolescence, where previous studies showed high rates of internalizing behaviors in girls [15]. These findings should be considered with caution and needs further investigation, because Italian adolescents previously reported as suffering from internalized or externalizing disorders, obtained through a DSM-IV assessment, was low: respectively 6,5% and 1.2% [8].

The most intriguing finding was the high prevalence rate in problems on *Competence scale* with opposite scores on *Activity* and *Social functions* between males and females. We speculate that CBCL *Competence Scale* does not strictly apply to the living framework of our sample, for example there is not such tradition as “clubs” for children. Other possible reasons may be the lack of structures for sports or juvenile aggregation in some areas of Campania or the possible negative influence of families on autonomy and extra familial ties.

Agreement between parents regarding behavioural or affective problems was quite high. As it could be expected, greater sensitivity of mothers for somatic and depressive symptoms could be easily explained by greater time spent with children. Moreover, problems related to the body and emotions areas represent two critical elements of mother-child primary relation.

Teachers seemed less able to identify problems than parents but for *Academic performance* or *School competence* subscale (problems that are more evident at school), where teachers compare students of the same age in learning and behaviour. Another explanation may be differences in tolerance of child behaviour and the studies to date showed discrepancies in parent and teacher ratings of social-behavioural functioning of children, as well as inter-raters concordance, especially for ADHD symptoms [16].

Parents reported attention problems much more frequently than teachers. This difference could ensue from a greater difficulty of being evaluated within a group or from a different meaning given by teachers (learning) and parents (other activities).

Main weakness of our study is the low generalizability of results because of possible selection bias due to peculiar characteristics of non responders and lack of consent of many eligible school. A similar responders rate was reported by Levi [17] in an Italian sample, while other studies in Europe showed a compliance rate from 50% to 70% [1-5]. However parental refusal (parents who do not return forms) was reported to be frequent in studies that use active consent procedures [18,19]. It is difficult to interpret the high rate of non-responders. As anecdote, we refer that parents’ main concern, at the school-meetings, was the possible stigma for students who should be rated clinical on CBCL. Furthermore, we observed poor interest in the goals of the research and wariness towards public institutions. It is worth noting that recently in Italy claims have been raised by several associations of families as regard school-setting researches on children/adolescents mental health. Cultural prejudices towards epidemiological studies and towards public sanitary institutions were also reported by other Authors [18,19]. Moreover, we report a high parents-teachers conflict during school meetings, regarding epidemiological data collection; this is probably due to lack of knowledge about the need of early detection of children with psychic difficulties.

Greater compliance of the mothers might be connected with the mothers’ traditional function of care-giving. Nevertheless, contribution to the study from both parents is greater than that of the mother alone, thus indicating the attitude to the child’s problems sharing in the parenting couple. The good compliance of teachers could be related to a greater knowledge of the instruments that were used and to expectations about the diagnosis and prevention programs for the problematic students.

Notwithstanding compliance limitations, the study provide useful preliminary information about the psychopathological profile in a population of 8 to 9 years old children, living in our region and it was strengthen by using both parents and teachers as informants.

Of course, CBCL screening does not estimate the prevalence of psychiatric disorders, that would require a second phase of psychiatric diagnosis based on DSM, but data on at-risk children could direct prevention programs, are relevant for services organization and need wider dissemination.

## Conclusion

The low compliance to present research revealed different factors that can limit future epidemiological studies and prevention programs involving children with mental disorders in Campania Region.

The prevalence of behavioural and affective disorders in the examined school-children in Campania Region is the same of the one reported in the rest of Italy. About 1 child out of 10 is at psycho-pathological risk, particularly regarding anxiety and depression. Difficulties in social and relationship competences area could be higher (4 children out of 10, as reported by parents). This datum deserves further studies and should be cautiously evaluated for the possible CBCL inadequacy about *Competences Scale.* Nevertheless, the result could indicate a risk condition for social and familial unfavorable factors in our region.

We are studying a further project for the screening/prevention of behavioural and affective problems in childhood and adolescence, that will involve children and family physicians.

## Endnote

^a^The second stage of the project (prevention) aimed to early detection and timely intervention of borderline subjects. The program consists of three counseling sessions for both children and parents at our University Clinic.

## Competing interests

The authors declare that they have no competing interests.

## Authors’ contributions

AG, CB and AP designed the study, interpreted data, drafted and revised the manuscript. SP, FS and GC collected data, drafted and revised the manuscript. SS and CG statistically analyzed data. All authors read and approved the final manuscript.
